# The impact of multiple non-pharmaceutical interventions on controlling COVID-19 outbreak without lockdown in Hong Kong: A modelling study

**DOI:** 10.1016/j.lanwpc.2021.100343

**Published:** 2021-12-18

**Authors:** Hsiang-Yu Yuan, Colin Blakemore

**Affiliations:** a Department of Biomedical Sciences, Jockey Club College of Veterinary Medicine and Life Sciences, City University of Hong Kong, Hong Kong SAR, China; b Department of Neuroscience, City University of Hong Kong, Hong Kong SAR, China; c Hong Kong Institute for Advanced Study, City University of Hong Kong, Hong Kong SAR, China

**Keywords:** Covid-19, Contact-tracing efficiency, Effectiveness of contact tracing, Confirmation delay, Non-pharmaceutical interventions, Epidemic modelling

## Abstract

**Background:**

The ‘third wave’ of COVID-19 in Hong Kong, China was suppressed by non-pharmaceutical interventions (NPIs). Although social distancing regulations were quickly strengthened, the outbreak continued to grow, causing increasing delays in tracing and testing. Further regulations were introduced, plus ‘targeted testing’ services for at-risk groups. Estimating the impact of individual NPIs could provide lessons about how outbreaks can be controlled without radical lockdown. However, the changing delays in confirmation time challenge current modelling methods. We used a novel approach aimed at disentangling and quantifying the effects of individual interventions.

**Methods:**

We incorporated the causes of delays in tracing and testing (i.e. load-efficiency relationship) and the consequences from such delays (i.e. the proportion of un-traced cases and the proportion of traced-cases with confirmation delay) into a deterministic transmission model, which was fitted to the daily number of cases with and without an epi‑link (an indication of being contact-traced). The effect of each NPI was then calculated.

**Findings:**

The model estimated that after earlier relaxation of regulations, *Re* rose from 0.7 to 3.2. Restoration of social distancing to the previous state only reduced *Re* to 1.3, because of increased delay in confirmation caused by load on the contact-tracing system. However, *Re* decreased by 20*.*3% after the introduction of targeted testing and by 17*.*5% after extension of face-mask rules, reducing *Re* to 0.9 and suppressing the outbreak. The output of the model without incorporation of delay failed to capture important features of transmission and *Re*.

**Interpretation:**

Changing delay in confirmation has a significant impact on disease transmission and estimation of transmissibility. This leads to a clear recommendation that delay should be monitored and mitigated during outbreaks, and that delay dynamics should be incorporated into models to assess the effects of NPIs.

**Funding:**

City University of Hong Kong and Health and Medical Research Fund.

**Research in context**

**Evidence before this study**

In our recent observational study of Hong Kong's third wave of COVID-19 [1] we found that re-tightening of social distancing measures alone failed to suppress the outbreak, because of decreasing efficiency of contact tracing and testing. We searched PubMed, bioRxiv, and medRxiv for articles published in English from 1 January 2020 to 31 March 2021, with the following keywords: (“2019-nCoV” OR “COVID-19” OR “SARS-CoV-2”) AND (“contact-tracing efficiency” OR “effectiveness of contact tracing” OR “confirmation delay” OR “contact-tracing delay” OR “testing delay”) AND (“model” OR “simulation”). Although 6 population-level modelling or simulation studies of COVID-19 predicted a relationship between confirmation delay and contact-tracing effectiveness, and suggested that confirmation delay should be minimized, none incorporated evidence of changing delay from a real outbreak. Without such consideration of changes in contact-tracing efficiency, the effects of interventions cannot be disentangled.

**Added value of this study**

We developed a model that incorporated variation in efficiency of tracing and testing in relation to the timing of NPIs. Through this modelling framework, we were able: i) to disentangle and quantify the effects of social distancing measures and policies that improve tracing and testing efficiency employed during an outbreak; ii) to identify the requirement of interventions that were able to suppress an outbreak; and iii) to reconstruct dynamics of true incidence.

**Implications of all the available evidence**

Our results demonstrate that the effect of each NPI employed during an outbreak can be accurately quantified, after incorporating variations in the delay. This approach to modelling can guide public health policies on outbreak control, providing an exit strategy without lockdown.

Alt-text: Unlabelled box

## Introduction

An important objective of epidemic modelling is to assess the impact of interventions and provide valuable insights for public health decision-makers.^2^ In this study, our intention was to quantify the effect of non-pharmaceutical interventions (NPIs) deployed during Hong Kong's ‘third wave’ of COVID-19, which was successfully suppressed without a lockdown.^1^ Hong Kong has adopted a ‘suppress and lift’ strategy,^3^ in which interventions are progressively strengthened when infections rise, and relaxed when they decline. These interventions include not only social distancing measures but also contact tracing, which aims to identify the close contacts of cases, who are immediately quarantined or self-isolated, and tested. If confirmed, they are isolated in hospital and reported as cases with the label ‘epi-link’ (epidemiological link).

Recent theoretical studies of COVID-19 have estimated what proportion of people have to be traced to reduce the effective reproduction number (*Re*) below 1, against the backdrop of various social distancing scenarios.^4^, ^5^, ^6^ Similarly, the impact of social distancing measures depends on the effectiveness of tracing and testing. For example, in Hong Kong, social distancing was relaxed during the period preceding the third wave, but re-introduction of measures even stricter than those before the relaxation did not stop expansion of the outbreak, because of decreased efficiency of the contact-tracing system.^1^

Variation in the load on contact tracing (e.g. the amount of people to be traced or tested) can affect its efficiency and hence the time between infection and confirmation (**confirmation time**). In the preceding paper, we analysed observational data from the third wave concerning the percentage of cases for which infection was not confirmed until after symptom onset (**confirmation delay**) and found that a rise in the proportion of such cases was associated with an increase in the number of recently confirmed cases.^1^ Presumably, a higher number of infected cases results in more close contacts and hence increases the load on the tracing and testing system.^5^ When tracing or testing capacity is limited or not sufficient, delays are likely to happen.

We have also shown that the growing load on contact tracing caused by increasing case number results in a vicious circle: increasing confirmation delay leads to more cases not traced, driving outbreak growth and further exacerbating the delay.^1^ Furthermore, the consequent change in confirmation time introduces errors into estimation of the timing of transmission and can therefore complicate public-health decision-making about when to strengthen or ease social distancing measures. This all highlight the need to know how to maintain the efficiency of contact tracing and testing, as well as how and when to tighten or lift social distancing regulations.

No previous model of COVID-19 has disentangled the effects of different NPIs deployed during an outbreak by taking account of variation in confirmation delay. We have incorporated evidence of the dynamics of delay during the third wave^1^ into a meta-population epidemic model, which provided an extensive and evidently accurate description of the pattern of transmission, and hence of the effects of NPIs. The model enabled us to quantify the contribution of each intervention in suppressing the third wave, and to formulate recommendations for the management of future outbreaks.

## Materials and methods

### Data sources

We retrieved data about epidemiological linkage and dates of symptom onset and confirmation for each local and imported cases of COVID-19 between 17 June and 15 August 2020 from the Hong Kong Centre for Health Protection.^7^ Confirmation delay was calculated as *Report date* minus *Date of onset* (for cases who were reported after symptom onset) from the official case report.

### Modelling delays in contact tracing and confirmation

**Figure 1:**
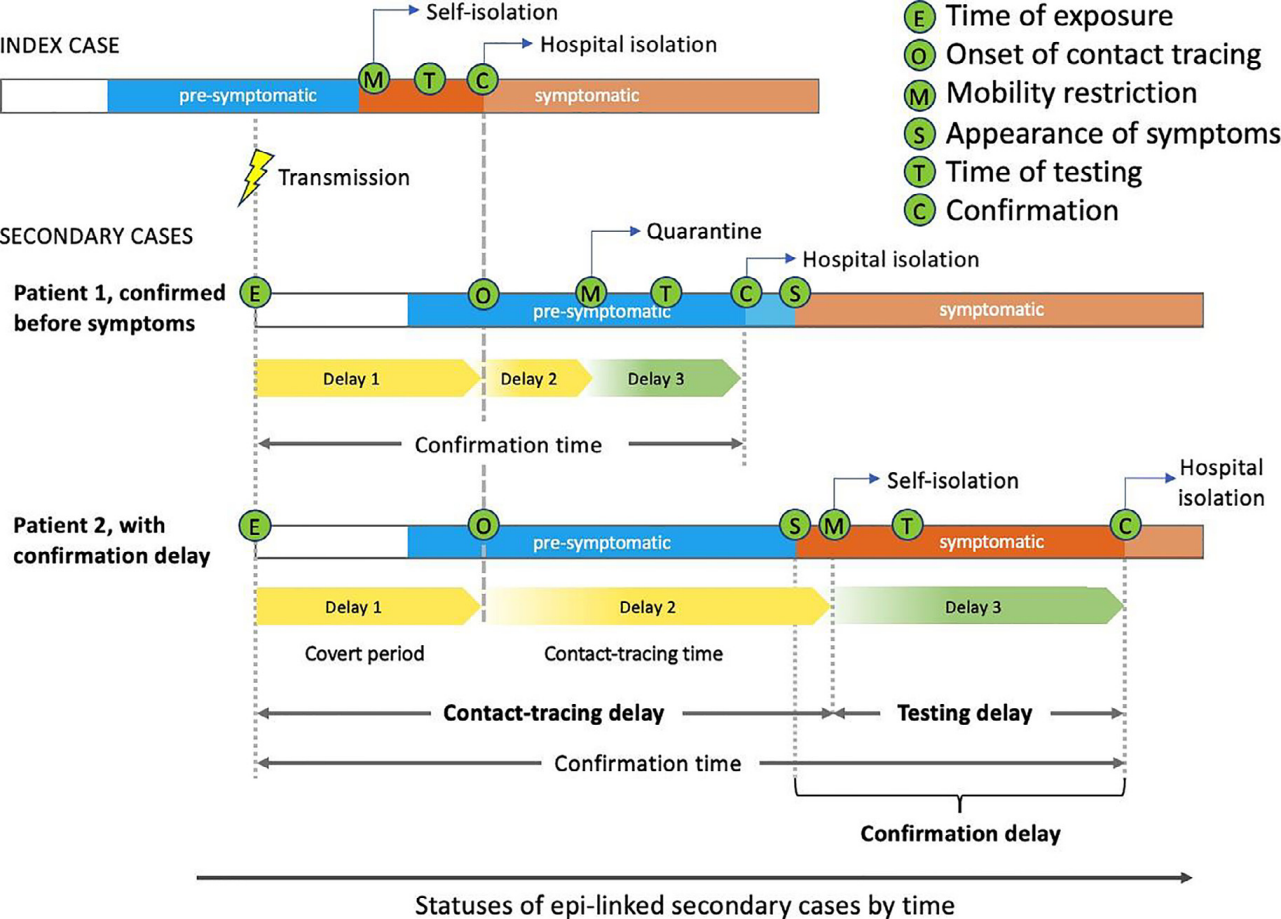
Schematic of the delays in contact tracing, testing and confirmation for epi‑linked cases. This diagram shows, schematically, the time-course of transmission of infection from an index case to two secondary cases: Patient 1, confirmed relatively quickly, before the appearance of symptoms; and Patient 2, confirmed after symptom onset. To simplify comparison of these two secondary cases, the time of exposure (E), the time from transmission to the onset of infectiousness (latent period; white bar), the pre-symptomatic transmission period (blue), and the symptomatic period (orange) have been made identical for the two. Light colours indicate that the cases are assumed not to be transmittable due to hospital isolation. The index case in the diagram is assumed to have been recognised and tested because of the appearance of symptoms, so there is an unavoidable period between transmission and confirmation, which is a delay (Delay 1) between exposure and the onset of contact tracing (E → O) for the secondary cases. We call this delay the **covert period**. Delay 2 (**contact-tracing time**), between the onset of contact tracing and the start of individual mobility restriction (O → M) is affected by contact-tracing capacity. **Contact-tracing delay** is the sum of these two delays, i.e. the time between exposure and the start of individual mobility restriction (quarantine or self-isolation) (E *→* M). Testing is performed during the period of mobility restriction, and confirmation of infection occurs some time later. The entire period between restriction and confirmation (Delay 3: M → C) is the **testing delay**, which is mainly affected by testing capacity. Each case is isolated in hospital as soon as infection is confirmed. The **confirmation time**, i.e. the time between exposure and confirmation (C), is the sum of the delays in contact tracing and testing. Patient 1 is traced and tested quickly and is confirmed before the onset of symptoms. But for Patient 2, the whole process is more prolonged. Delay 2 (contact-tracing time) is longer and this individual is already symptomatic when traced and mobility restriction (called self-isolation because it is after symptom onset) is imposed. More time is also taken for testing and confirmation (Delay 3; testing delay). In cases like this, confirmed after the appearance of symptoms, we call the time between symptom onset and confirmation the **confirmation delay** (S *→* C).

This modelling study is based on our analysis of observational data from the third wave,^1^ which revealed the relationship between the load on contact tracing and testing and their efficiency. In order to capture the dynamics of local cases, both with and without an epi‑link, we extended a classic Susceptible-Exposed-Infectious-Recovered (SEIR) model to incorporate quarantine, isolation and the variations in confirmation time, including contact-tracing delay and testing delay (see Figure S1 and Supplementary Material). We incorporated a two-step process (i.e. contact tracing and testing), for which inefficiency leads to **confirmation delay** (i.e. time between symptom onset and confirmation) (Figure 1). In the first step, an index case was confirmed as COVID-19 positive, and contact-tracing delay in secondary cases was calculated. Increased delay (as for Patient 2 in Figure 1) could be caused by a confirmation delay for the index case (covert period) and/or delays in contact-tracing time. Because we assumed that cases were isolated immediately after confirmation, this delay is equivalent to “delay from onset to isolation”, as used by Hellewell et al. and other previous studies.^4^^,^^8^ The magnitude of the confirmation delay depends not only on contact tracing and testing capacity but also on the duration of the covert period.

Mobility restriction (i.e. quarantine or self-isolation) for a contact case started at the time that the contact was successfully traced. The contact-tracing time, *f* (*κ_c_, Tshd_c_*), was determined by a capacity threshold *Tshd_c_* (measured in number of cases), an indicator of the load of contact tracing *Cases_c_*(*t*), and the range of delay specified by minimum and maximum delay (i.e. *MinDelay_c_* and *MaxDelay_c_*(*t*)):

(1) $$f\left(\kappa_{c},\;\mathrm{Tsh}d_{c}\right)=\mathrm{MinD}\mathrm{ela}y_{c}+\left(\mathrm{MaxD}\mathrm{ela}y_{c}\left(t\right)-\mathrm{MinD}\mathrm{ela}y_{c}\right)\left(1-e^{-\left(\mathrm{Case}s_{c}\left(t\right)-\mathrm{Tsh}d_{c}\right)/\kappa_{c}}\right)$$

where *Cases_c_*(*t*) is the number of cases in quarantine and self-isolation at time *t* and *κ_c_* is a capacity scale factor. In our preceding study,^1^ we found that the number of recently confirmed cases was a simple indicator of the load on tracing and testing. Now, in the modelling framework, we specified this indicator more precisely. When the case number was higher than the threshold, contact-tracing time began to increase, approaching a maximum value, *MaxDelay_c_*(*t*).^1^ When the case number was lower than the threshold, we set contact-tracing time to *MinDelay_c_. MaxDelay_c_*(*t*) can be decreased by interventions, indicating a reduction of delay. Contact-tracing delay (*T_qr_*) was then defined as the sum of the contact-tracing time and covert period, such that *T_qr_* = *f* (*κ_c_, Tshd_c_*) + *k*⋅*g*, where *g* is a function representing testing delay (see below) and *k* is a factor to determine the contribution from the index case (covert period). The minimum delay in contact tracing *MinDelay_c_* represents an irreducible time for contact tracing.

In the second step, testing delay, from self-isolation or quarantining until confirmation, was calculated for contact-traced individuals. We assumed that all confirmed cases were immediately hospital-isolated. Therefore, the expected time between the start of mobility restriction and hospital isolation was taken as testing delay (*T_hos_* = *g)*. This is determined by a capacity threshold *Tshd_t_* (measured in number of cases) and a capacity scale factor *κ_t_*:

(2) $$g\left(\kappa_{t},\mathrm{Tsh}d_{t}\right)=\mathrm{MinD}\mathrm{ela}y_{t}+\left(\mathrm{MaxD}\mathrm{ela}y_{t}\left(t\right)-\mathrm{MinD}\mathrm{ela}y_{t}\right)\left(1-e^{-\left(\mathrm{Case}s_{t}\left(t\right)-\mathrm{Tsh}d_{t}\right)/\kappa_{t}}\right)$$

where *Cases_t_*(*t*) here is the number of cases in quarantine, self-isolation and hospital isolation at time *t*. Because all these cases required testing, this number corresponds to the load on testing. When the case number was higher than the threshold, testing delay began to increase, approaching a maximum value, *MaxDelay_t_*. When the case number was lower than the threshold, we set testing delay to *MinDelay_t_*. We assumed that hospital isolation ensures perfect isolation effects but cases in quarantine and self-isolation may still contact others with a lower contact rate (see Supplementary Material). In some circumstances, delay in hospital isolation of infected cases may leads to a late start of contact tracing. Because when patients were staying at home and hard to reach, the covert period became longer. The values of these parameters are obtained after model fitting (Table S3). Detailed approaches are described in Supplementary Methods.

For individuals who were back-traced after symptom onset, rather than contact-traced from a preceding case, confirmation delay was taken to be testing delay (see Supplementary Material). Amongst all infectious individuals who were not contact-traced, we assumed that a fixed fraction *p*1 developed symptoms and were eventually isolated in hospital. The remaining asymptomatic cases recovered without being detected. We incorporated these delays into our meta-population model framework, which included the seeding effects from undetected imported cases amongst exempted visitors^1^ (see Supplementary Material).

### Modelling the consequences of confirmation delay

**Figure 2:**
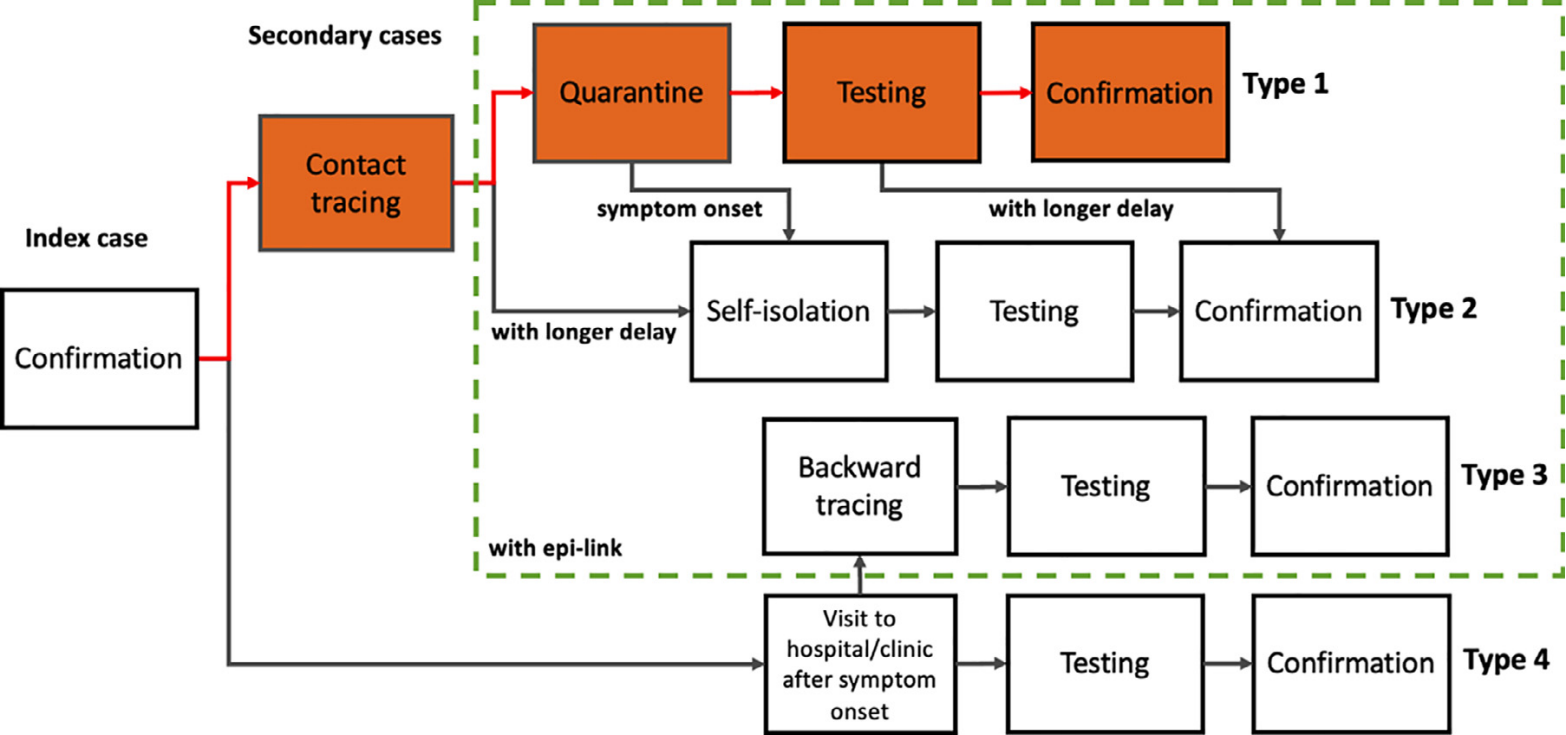
Schema of four types of secondary cases, taking account of delays in contact tracing and testing. **Type 1: Contract-traced without confirmation delay** (infection confirmed before symptom onset). Red arrows and orange boxes represent the critical flow to confirm a positive secondary case without confirmation delay. The suspected case is quarantined as soon as identified by contact tracing and isolated in hospital immediately after confirmation. **Type 2: Contact-traced with confirmation delay**. The case is detected and confirmed, with an epi‑link, but delay in contact tracing and/or testing causes confirmation to occur after symptom onset. **Type 3: Backward contact-traced**. These individuals report to hospital or a clinic with symptoms rather than being identified by contact tracing, but they are subsequently backward-traced to a source. They have a confirmation delay. Types 1, 2 and 3 (in dashed green rectangle) all have an epi‑link. **Type 4: No epi‑link**. These individuals visit a hospital or clinic with symptoms and are subsequently confirmed, but with a confirmation delay. No epi‑link is found, even by backward tracing.

Confirmation delay resulted in four types of cases (Figure 2). Success in tracing contacts from a confirmed index case had two possible consequences:

1. **Contact-traced without confirmation delay**. Exposed individuals who were traced before symptom onset were quarantined and tested. Some of them were confirmed before symptoms, if the testing delay was short.
2. **Contact-traced with confirmation delay**. Longer testing delay led to some quarantined individuals developing symptoms before confirmation. In addition, because of delays in contact tracing, some individuals were traced and self-isolated and then tested, after they developed symptoms.

Cases not epi‑linked by normal ‘forward’ contact tracing, fell into two groups:

3. **Backward contact-traced**. For some cases, originally not belonging to any close contacts, a contact to an earlier case or infection source was found by ‘backward’ tracing.^9^
4. **No epi‑link**. In many cases, especially during growth of the outbreak, no epi‑link was found to a preceding case.

Many cases were identified after notification in symptomatic surveillance, or through routine testing, rather than by ‘forward’ contact tracing. (Most of them were tested because they had symptoms). Hence, backward tracing began after symptom onset. About 1.7 million people were tested in a mass-screening programme, but this was not introduced until 1 September 2020, after the major decline of the outbreak.^10^

The output of the model (see Supplementary Material and Figure S1) is the daily number of these four types of cases. Using these numbers, we were able to estimate two useful indicators of reduced effectiveness of contact tracing and testing:

1. **Contact-tracing inefficiency**, indicated by the proportion of all cases in which no epi‑link was established (case type 4);
2. **Tracing and testing inefficiency**(also called**confirmation inefficiency**), specified as the fraction of epi‑linked cases with confirmation delay (case types 2 and 3, as a percentage of case types 1, 2 and 3).

### Quantifying the effects of interventions

We incorporated the causes of delays in tracing and testing (Figure 1) into an extended SEIR meta-population model (See Supplementary Material) and considered their consequences for reported cases (Figure 2), amongst which contact-traced cases were labelled with an ‘epi-link’. The model considered the seeding of local transmission by imported cases. The number of undetected imported cases was calculated in the preceding study (Figure S1 in^1^).

The individual effects of social distancing measures and policies that affect efficiency of contact tracing or confirmation were disentangled through modelling, even though the periods of these interventions overlapped. This enabled the model to project the dynamics of cases in scenarios in which individual interventions were excluded.

The expected daily numbers of confirmed cases that were traced and un-traced were projected from the model and compared with the daily numbers of reported cases with and without an epi‑link. Using the next-generation matrix approach, *Re* can be calculated from the posterior distribution of epidemiological parameters obtained after model-fitting (see Supplementary Material). We calculated the effect of each intervention on *Re*, i.e. the relative reduction in *Re* resulting from one particular intervention when it was introduced. Note that the effect of targeted group testing (TT) is conditional on the case number, determined by preceding social distancing regulations.

We did not explicitly fit the model to the recorded delays in the confirmation of cases. Instead, in order to validate the model, we compared the model-produced delays in confirmation of cases with the reported epi‑linked cases that had confirmation delay. We investigated whether the model-produced delays (contact-tracing inefficiency and confirmation inefficiency) resembled the shape of observed data by counting how many days of the observed data (moving-averaged) located within 95% credible interval. Then we compared the outputs of this model with those of the same model framework but without incorporation of variations in contact-tracing delay and testing delay (the ‘reduced’ model) (see Supplementary Material).

### Calculating effective reproduction number

We calculated *Re* using the next-generation matrix approach after obtaining the posterior distributions of the model parameters.^11^ We obtained the transmission matrix *T* and the transition matrix *S*. An element in each matrix represents the average number of cases in a particular category (specified by the row) transmitted or transited from the same or different categories of cases (specified by the column). *R_e_* was calculated as the first eigenvector of (*TS*^−1^). See Supplementary Material for the specification of the matrices.

### Role of the funding source

The sponsor of the study had no role in study design, data collection, data analysis, data interpretation, or writing of the report. The corresponding author had full access to all the data in the study and had final responsibility for the decision to submit for publication.

## Results

To investigate how the combination of NPIs contributed to suppression of the outbreak, we modelled the separate effects of social distancing interventions and changes in the efficiency of tracing and testing, by taking account of the relationship between the dynamics of transmission and confirmation delay.

### Impact of interventions on *Re*

**Figure 3:**
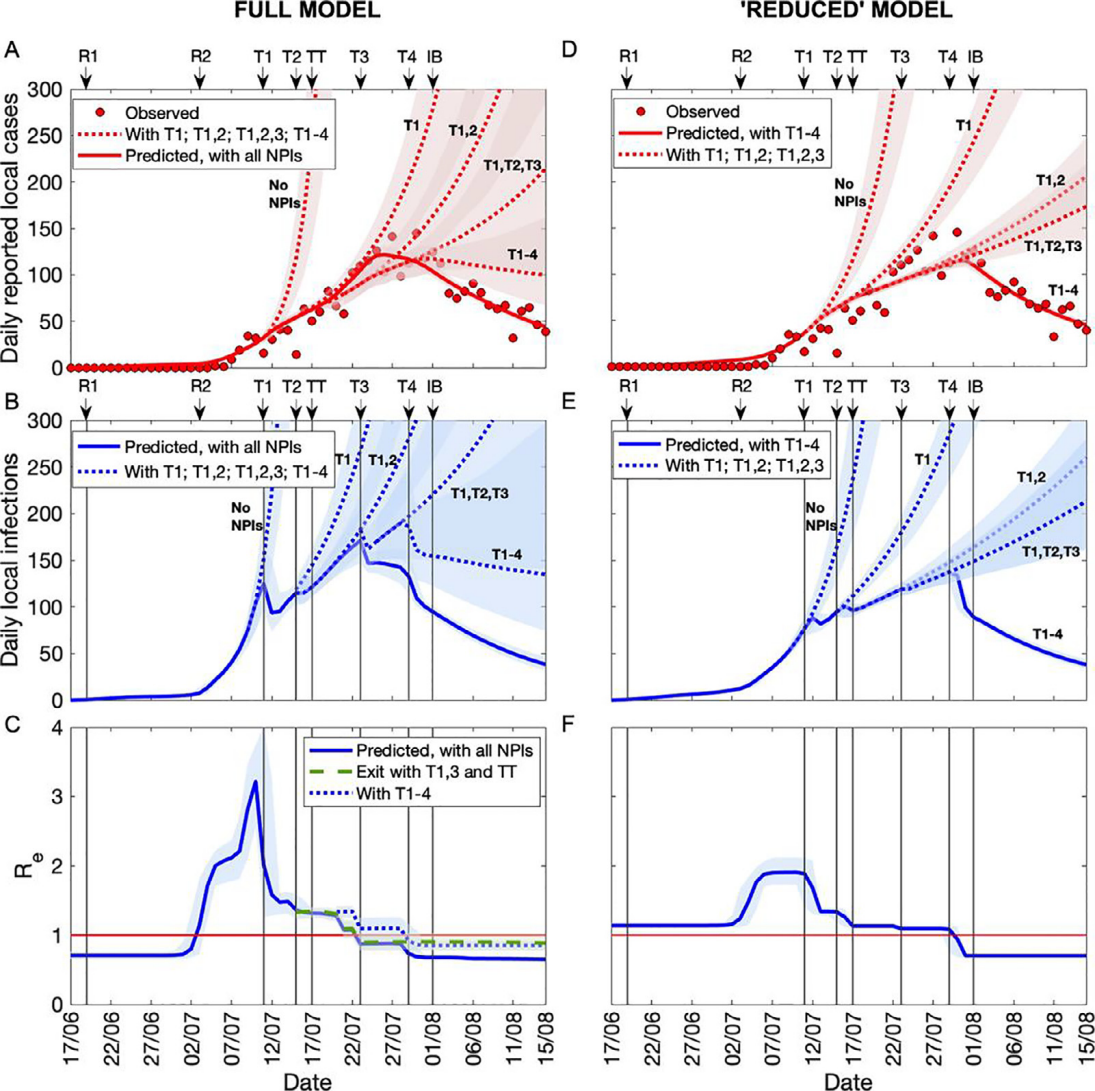
COVID-19 transmission dynamics in relation to the introduction of NPIs, derived from the full model (A, B, C) and from the ‘reduced’ model (without incorporation of delay dynamics) (D, E, F). The arrows above the upper abscissa and the vertical lines indicate the relaxation of social distancing regulations on 19 June (**R1**) and 3 July (**R2**), and the dates of the various NPIs introduced during the outbreak, using abbreviations from Table 1. (A) Actual daily reported local cases (red circles) and daily cases projected by the full model, taking account of all NPIs (red continuous line). The red dashed lines indicate the predicted mean values of daily cases with various combinations of interventions. **No NPIs** shows the predicted exponential growth if no new NPIs had been introduced. The other red dashed curves show the projected effects of T1 alone and of various combinations of social distancing measures (**T1,2; T1,2,3**; and **T1–4**) but without the interventions aimed at increasing contact-tracing efficiency: targeted testing (**TT**) and boosting of isolation capacity (**IB**) (see Table 1). (B) The continuous blue curve plots the model-derived numbers of daily local infections, assuming all NPIs. The dashed curves show the projections with various limited combinations of NPIs, as in (A). (C) The model-derived effective reproduction number *Re*. The solid blue line plots the projected curve, taking account of all NPIs. The green dashed line shows the projected dynamics of *Re* with only T1, T3 and TT, i.e. the minimum combination of interventions needed to suppress this outbreak against the back-drop of strengthened contact-tracing efficiency. The blue dotted line plots the projected dynamics with all social distancing measures alone, without TT. *Re* of 1.0 is indicated by a horizontal red line. All values were estimated from 400 random samples from the posterior distributions. 95% credible intervals are shown in light colours. (D), (E), (F), show results, plotted in the same way, for the ‘reduced’ model (without delay dynamics).

Our model derived the true daily infection rate and *Re* along with the dynamics of reported cases, and quantified the contribution of each individual NPI (Figure 3A, B, C). The model assumed that the outbreak was triggered by undetected imported cases (infected individuals exempted from quarantine and not tested on arrival), and amplified by relaxations of social distancing, on 19 June and 3 July (R1 and R2; see Table 1). After the outbreak began, social distancing measures were strengthened four times (Table 1), sequentially reducing the maximum number of people who could gather in public places or restaurants from 50 to 8, then 4, then 2, as well as mandating mask-wearing in all indoor public places.

**Table 1:** Dates of relaxation of social distancing measures and implementation of significant public health interventions. Effects (relative reduction) on *Re* are listed for NPIs deployed during the outbreak.

	Intervention	Date (2020)	Details	Effects (95% CI)
**R1**	Relaxation of regulations	19 June	Maximum number of people permitted to gather in public places raised from 8 to 50.^21^	–
**R2**	Further relaxation	3 July	Maximum number in places of entertainment raised from 50% to 80% of capacity.^22^	–
**T1**	Tightening 1 (Gathering ban)	11 July	Maximum number of people gathering at catering premises reduced to 8. Maximum number in places of entertainment reduced from 80% to 60% of capacity.^23^	60.7% (52.1–69.0%)
**T2**	Tightening 2 (Gathering ban)	15 July	Maximum number of people gathering in public places and at restaurant tables reduced to 4. Maximum number in places of entertainment reduced to 50% of capacity. Closure of schools (from 13 July).^24^	13*.*9% (3*.*4–20*.*8%)
**TT**	Targeted testing services	17 July∗	Introduction of testing for specific target groups.^25^	20*.*3% (14*.*1–25*.*9%)
**T3**	Tightening 3 (Face-mask rule)	23 July	Mandatory mask-wearing extended from public transport∗∗ to all indoor public places.^26^	17*.*5% (1*.*9–36*.*4%)
**T4**	Tightening 4 (Gathering ban)	29 July	Reduction of maximum number gathering in public places from 4 to 2. No service in restaurants in the evenings. (A total ban on the first day was immediately lifted).^27^	22*.*5% (6*.*1–35*.*1%)
**IB**	Isolation capacity boosting	1 August	Community treatment facility opened∗∗∗ at Asia World-Expo site.^28^	2*.*0% (0*.*1–7*.*4%)

∗ The first phase of targeted testing (for care home staff) started on 14 July, but it yielded only one case. The major part of the programme, starting on 17 July, involved testing nearly 150,000 taxi drivers and restaurant staff, revealing 34 cases. 17 July was a Friday and the first batch of specimens was collected after the weekend, on 20 July.^29^ Over the following 2 months, testing was then rolled out for other groups, including property management staff, market workers, transport workers, and residents of housing estates with transmission clusters. In all the phases that started during the period of this study, 76 cases were discovered from 414,085 tests, but the bulk of cases were found in phases launched between 17 July and the end of July.^30^

∗∗ From 13 July, it was mandatory to wear face-masks on public transport. However, this is unlikely to have had significant impact because masks were already almost universally worn on public transport, and also in the streets.

∗∗∗ On 23 July additional community isolation facilities were opened.^17^ However, this was a modest initiative, which appears to have had little impact on tracing and testing efficiency.

**Fig. 4:**
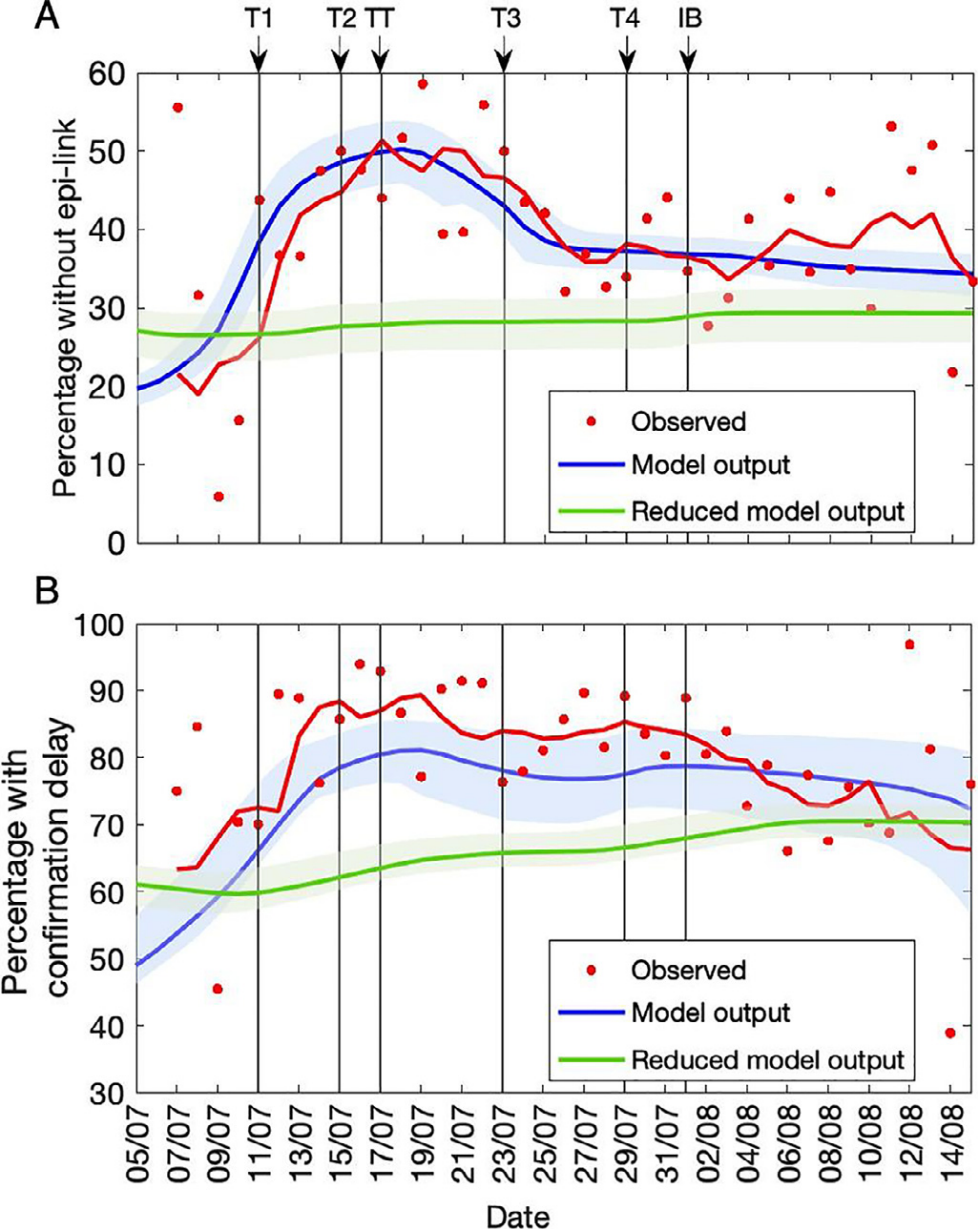
Comparisons between actual values and model projections for two indicators of reduced effectiveness of tracing and testing, namely: (A) **contact-tracing inefficiency** (i.e. percentages of all local cases without an epi‑link) and (B) **tracing and testing** (or **confirmation) inefficiency** (i.e. proportion of epi‑linked cases with confirmation delay); In both graphs, red circles represent the observed proportion each day and the red curve is a moving average (5-day window, centred at day 3). The blue curve represents the model-derived output, while the green curve is the output of the ‘reduced’ model that did not incorporate the dynamics of delay. Credible intervals were generated by 400 repeated samplings from the posterior distributions.

The model estimated that, immediately after the second relaxation of social distancing (R2) on 3 July, before the first case in the outbreak was reported, the effective reproduction number, *Re*, rapidly increased from 0.7 to greater than 2, following an assumed logistic curve (Figure 3C). Starting on 9 July, there was a second sharp increase of *Re* to 3.2, before the first intervention, T1, on 11 July (Figure 3C). This second increase was caused by the effect, in the model, of decreasing efficiency in contact tracing and testing because the number of reported cases was larger than the capacity thresholds of contact tracing and testing (both about 20 cases; see Methods and Table S3). E.g. the average cumulative number of reported cases within 10 days had reached 110 on 10 July, when the peak of *Re* occurred. This reduction is reflected in the model's prediction of rising trends in the proportion of both un-traced cases and those with confirmation delay (Figure 4).

The model captured the rapid increase of local cases during the first three weeks of the outbreak (Figure 3A) and revealed the impact of Tightening 1 (T1: maximum gathering number reduced from 50 to 8 persons). Although this single intervention imposed virtually the same restrictions as were in place before R1, it reduced *Re* only to 1.3 (relative reduction: 60.7% (95% CI 52*.*1–69%)) (Figure 3C and Table 1). The model suggested that this failure to reverse completely the trend of local spreading resulted from the increasing proportion with confirmation delay (Figure 4). Tightening 2 (T2: reducing the maximum gathering from 8 to 4) had little additional impact (13*.*9% (95%CI 3*.*4–20*.*8%)). Neither T1 nor the combination of T1 and T2 prevented the outbreak from growing exponentially. For a week, *Re* remained at about 1.3 (Figure 3C).

The extreme measures introduced on 29 July (T4) had a substantial effect on *Re* (22*.*5% (95%CI 6*.*1–35*.*1%)), and the daily number of confirmed cases began to decline around that time. However, the modelling results show that the daily rate of infections had already started to decline (Figure 3B) mainly because of targeting group testing (TT; 20*.*3% (95%CI 14*.*1–25*.*9%)) and the face-mask rule (T3; 17*.*5% (95%CI 1*.*9–36*.*4%)), which were introduced several days earlier. T3 and TT, combined with the effect of the early gathering restrictions (especially T1), reduced *Re* to 0.9 around 23 July (Figure 3C). The outbreak had effectively been suppressed by the NPIs introduced before T4.

The value of incorporating dynamics of contact-tracing efficiency is amply demonstrated by the result of re-running the model without incorporating variation in efficiency (see Supplementary Material). While this simplified ‘reduced’ model predicted the overall time-course of observed cases fairly well (Figure 3D), the striking correlation between NPIs and infection rate seen in Figure 3B was eroded (Figure 3E). The projected dynamics of *Re* showed a single rise before the outbreak (Figure 3F) and failed to demonstrate the lack of reversibility resulting from load on the contact-tracing system: *Re* returned to virtually its initial value, slightly above 1.0, after T1 and T2. In the ‘reduced’ model, T3, during the plateau, had almost no impact on *Re* or infections, while T4 had a dramatic effect (Figure 3E, F). The decline in infections before T4, revealed by the full model (Figure 3B), was unseen.

The prediction of daily infections from the ‘reduced’ model (Figure 3E) was more similar in shape to that of daily case numbers (Figure 3D) than for the outputs of the full model (compare Figure 3A,B). The dynamics of infection (Figure 3E) and of *Re* (Figure 3F), in relation to NPIs, were substantially different from those of the full model (Figure 3B,C).

### Impact of minimizing delay on transmission dynamics

The dotted curve in Figure 3C shows the estimated *Re* profile for all social distancing interventions but without the improvements in tracing and testing efficiency (TT and IB). Confirmation time would have grown more rapidly, hence extending and flattening the plateau, causing the projected daily case number to be temporarily lower than the reported numbers (dotted line, T1–4, in Figure 3A).

The model captured the decrease in efficiency of contact tracing during epidemic growth and the restoration of efficiency about 5 days after targeted group testing (TT) was introduced (Figure 4A). Percentage without epi‑link reduced from 47% to 37% within a week. Boosting of isolation capacity (IB) appeared to have only a minor effect on efficiency, and therefore on *Re* (Table 1). The validity of the model is shown by the fact that it also captured the proportion with confirmation delay amongst cases with an epi‑link (Figure 4B). This increased rapidly when the daily case number was growing and remained at a high level until a few days after the introduction of TT. In contrast, the ‘reduced’ model comprehensively failed to predict the time-courses of either epi‑linkage or confirmation delay (Figure 4). The ‘reduced’ model only successfully captured the observed smoothed curve in 2 out of 40 days (within 95% credible interval) whereas the full model captured 25 days in epi‑linkage. Similarly, for confirmation delay, 7 out of 40 days were captured from the ‘reduced’ model while 30 out of 40 days were captured from the full model. Note that the two indicators from the ‘reduced’ model changed slightly over time because of the transient dynamics of the relevant statuses of the four types of cases, even though confirmation time was fixed.

Improvement in the efficiency of tracing and testing together with the strengthened social distancing measures (T1–3), successfully curtailed the growth of the outbreak. The stringent social distancing measures in T4 lowered the number of cases, hence reducing the load in trace-and-test and further improving the efficiency of tracing and testing (Figure 4B). Together, these NPIs successfully suppressed the third wave.

## Discussion

Knowledge of the dynamics of infection and the impact of interventions is crucial for public-health decision-making. For the first time, we have enhanced an epidemic model with a wealth of observational data on variation in contact-tracing efficiency during a substantial outbreak. This improved the estimation of the pattern of infection dynamics and the separate impact of individual interventions (Figure 3A-C). We validated the model by showing that the results of its simulation matched the time-course of the proportion of traced cases with confirmation delay (Figure 4B).

During Hong Kong's third wave, the early social distancing regulations, T1 and T2, did not succeed in reducing *Re* to its original level, even though they imposed stricter controls than before the initial relaxation (Table 1).^1^ This hysteresis in the effect of social distancing measures was mainly due to decreasing efficiency of tracing and testing during the expansion of the outbreak.

We found that the improvement in contract-tracing and testing efficiency resulting from testing at-risk groups (TT) played a crucial role in enabling social distancing measures to suppress the outbreak. Our estimates confirm the suggestion that testing selected groups, regardless of whether they have symptoms, can decrease transmission of the virus.^12^

There has been a lively debate about the effectiveness of face-masks, with disparate conclusions.^13^ Our results suggest that mandating the wearing of face-masks in all indoor public places (T3) was associated with a significant effect on transmission. However, this might have been partly due to other changes in behaviour precipitated by the rapid increase in case numbers. Moreover, mask-wearing, by heightening risk perception, might itself have affected social interactions.

Modelling that incorporated variation in efficiency of contact tracing gave us a better understanding of what caused rapid growth of the outbreak and how it was suppressed. We were able to correlate the NPIs with the dynamics of the outbreak and of confirmation delay, and hence to rank the most effective interventions, in order of their impact on *Re*:

1. Strengthening of the gathering ban, especially the reduction in the number allowed to meet from 50 to 8 (T1): 60*.*7%;
2. Targeted testing (TT), which substantially reduced contact-tracing delay and testing delay: 20*.*3%;
3. The requirement to wear face-masks in public places (T3) and possible associated behavioural changes: 17*.*5%

The accuracy provided by incorporating the dynamics of contact-tracing efficiency is vividly illustrated by comparison with results from the same model excluding variation in confirmation delay (Figure 3D-F, Figure 4). It is gratifying to see that important features of transmission dynamics and of the impact of NPIs, seen in the output of the full model, are not revealed by this ‘reduced’ model. For instance, it predicts that the early interventions, T1 and T2, should have restored *Re* virtually to 1; it fails to detect the significant effect of T3; and it over-estimates the impact of the extreme social distancing measures in T4.

### The importance of monitoring and minimizing confirmation delay

While the importance of tracing and testing is universally acknowledged, policy-makers have been lacking clear guidance on optimizing strategies to improve the efficiency of tracing.^14^^,^^15^ There have been efforts to use simulation to estimate the proportion of cases that must be traced in order to reduce *Re* below one.^5^^,^^6^^,^^16^ These are important but unfortunately, the number of undetected cases is usually unknown, and the significant proportion of asymptomatic cases complicates the estimation of the number, as well as hindering the performance of contact tracing.^17^ Our study clearly demonstrates that during the third wave, confirmation delay amongst traced cases affects *Re*. One assumption we made in modelling the impact of confirmation delay on *Re* is that most of the cases are soon hospital isolated following their confirmation. This happens in Hong Kong, which certainly ensures isolation effectiveness. This supports a clear recommendation that confirmation delay should be closely monitored during outbreaks, and measures should be introduced to prevent increasing delay.

Targeted group testing increased the speed and completeness of identification of cases amongst close contacts in transmission clusters and other at-risk groups, and presumably played a part in increasing the proportion of epi‑linked cases and controlling growth of the outbreak. Accurate identification of at-risk groups for targeted testing, using contact data and/or mobility data,^18^ is essential.

### Exit without lockdown

Finding strategies to avoid or manage recurrent outbreaks without damaging lockdowns, while we wait for the global roll-out of effective vaccines, is a major challenge.^19^^,^^20^ The present study illustrates how the tightening of social distancing measures, combined with strengthened tracing and testing, succeeded in suppressing Hong Kong's third wave. This suggests that, even when case numbers are very low, social distancing should be relaxed with great caution, to prevent a sudden rise in transmissions that can temporarily overwhelm the capacity of contact tracing and testing. It also highlights the importance of incorporating measures of tracing and testing efficiency into modelling, to give more reliable estimates of the dynamics of transmission, and hence to provide more accurate assessment of public health risk and interventions.

## Author contributions

HY and CB designed the study. HY analysed and modelled the data. HY and CB wrote the paper

## Editor note

The Lancet Group takes a neutral position with respect to territorial claims in published maps and institutional affiliations.

## Data sharing

The source code and data for the work is available at https://github.com/hy39/npi_hk_wave3.

## Declaration of interests

All authors declare no competing interests.

## Supplementary materials

Image, application 1
