# Supplementary material for: The impact of multiple non-pharmaceutical interventions on controlling COVID-19 outbreak without lockdown in Hong Kong: A modelling study
Source: Lancet Reg Health West Pac. 2021 Dec 18;20:100343. doi: 10.1016/j.lanwpc.2021.100343 (PMC8683252; doi:10.1016/j.lanwpc.2021.100343)
Supplement: Supplementary file 1 [file mmc1.pdf]

Supplementary Material

The impact of multiple non-pharmaceutical interventions on controlling COVID-19 outbreak without lockdown in Hong Kong: a modelling study

Hsiang-Yu Yuan[1,*], Colin Blakemore[2,3]

1 Department of Biomedical Sciences
Jockey Club College of Veterinary Medicine and Life Sciences
City University of Hong Kong
Hong Kong SAR, China

2 Department of Neuroscience
City University of Hong Kong
Hong Kong SAR, China

3 Hong Kong Institute for Advanced Study
City University of Hong Kong
Hong Kong SAR, China

*Correspondence to Hsiang-Yu Yuan: sean.yuan@cityu.edu.hk

Supplementary Methods

Our aim was to quantify the effects of the NPIs implemented during the 'third wave' COVID-19 outbreak in Hong Kong, which was likely triggered by undetected imported cases after the relaxation of social distancing measures. We first estimated the undetected imported cases among those who were exempt from quarantine at entry (see section *Incorporating undetected imported cases* below). Second, we developed an SEIQR-D meta-population model that includes dynamics from imported and local cases (section *SEIQR-D meta-population model*) and incorporates variations in confirmation delay (section *Incorporating variations in confirmation delay*). During the outbreak, the contact rates were changed progressively after each tightening of social distancing measures (section *Incorporating effects of Tightening Social Distancing Measures*). After model-fitting using MCMC (section *Parameter estimation*), the transmission dynamics were produced, along with two indicators of contact-tracing inefficiency: the proportion of cases without an epi-link and the proportion of contact-traced cases with confirmation delay (section *Mapping the model to cases with epidemiological links*). The effects of NPIs were then disentangled and quantified after calculating the effects (relative reduction) on *Re* (section *Calculating effective reproduction number*). The model output was compared with a 'reduced model' that did not take into account the variation in confirmation delay (section *Model-fitting without variation in confirmation delay*).

Incorporating undetected imported cases

In the preceding paper [1], we derived the number of undetected imported cases:

$$\#Undetected\, imported\, cases(t) = \frac{\rho}{1-\rho} \cdot (\#Reported\, imported\, cases(t)) \tag{1}$$

where ρ is the product of the ratio of the number of exempted visitors to the total number of visitors and the percentage of asymptomatic cases. We assumed that the percentage of asymptomatic cases was 17% [2].

SEIQR-D meta-population model

We assumed that the new emergence of COVID-19 was initiated by undetected imported cases. Therefore, a meta-population model was developed in order to consider the virus spreading from foreign places to Hong Kong with infected visitors, some of whom were exempt from compulsory quarantine at entry. First, we constructed the dynamics of imported cases:

$$\frac{dE_{imp}}{dt} = smooth(Imp) - \left(\frac{1}{\tau} + \frac{1}{T'_{qr}}\right) E_{imp} \tag{2}$$

$$\frac{I_{ud,imp}}{dt} = \frac{\rho}{1-\rho} \cdot smooth(Imp) \cdot (1 + \Lambda \times \frac{t}{30}) + \frac{1}{\tau} E_{imp} - \frac{1}{T_C} I_{ud,imp} \tag{3}$$

$$\frac{dQ_{E,imp}}{dt} = \frac{1}{T'_{qr}} E_{imp} - \frac{1}{\tau} Q_{E,imp} \tag{4}$$

$$\frac{dQ_{I,imp}}{dt} = \frac{1}{\tau} Q_{E,imp} - \frac{1}{\tau} Q_{I,imp} \tag{5}$$

$$\frac{dR_{imp}}{dt} = \frac{1}{T_C} (I_{imp} + I_{ud,imp} \tag{6}$$

where E_{imp}, $I_{ud,imp}$, $Q_{E,imp}$, $Q_{I,imp}$, R_{imp} represent the number of imported cases in different statuses, i.e. exposed, undetected infectious, quarantined exposed, quarantined infectious and recovered, respectively (see Table S1). $\frac{\rho}{1-\rho}$, which allows us to estimate the number of undetected imported cases, was set to be 0.1 following the calculation in our preceding study [1]. Imp is the number of reported (confirmed) imported cases per day [3]. $smooth(Imp)$ is the smoothed estimate of the incidence Imp. We used a 5-day moving average of Imp in order to reduce daily variation. $smooth(Imp)$ determines the number of exposed imported cases E_{imp} each day. Λ, the rate of change per day in the number of exempted cases, was 0.6%, calculated from the data from the Immigration Department [4]. $\frac{1}{T'_{qr}}$ was set to be 20, enabling nearly all the exposed imported cases E_{imp} to be immediately quarantined as $Q_{E,imp}$ (97%).

To build the disease transmission model, we first extended a classic SEIR model to include quarantine, self-isolation, and contact tracing [5]. Then

we further broadened this model by incorporating the variations in confirmation delay resulting from the changes in load on contact tracing and testing. This model was designated SEIQR-D to indicate that effects of quarantine (Q) and the variations in **confirmation delay**(D) were taken into account. The model can produce the dynamics of local cases, with and without an epidemiological-link (epi-link) and the proportion of contact-traced cases, with and without confirmation delay.

After local transmission was seeded by the undetected imported cases $I_{ud,imp}$, infected cases that were contact-traced were put under the mobility restriction in one of the three ways:
i) quarantined (at home or a designated facility), for cases identified as a contact before showing symptoms;
ii) self-isolated (at home or a designated facility), for cases identified as contact after symptom onset or cases who developed symptom while in quarantine;
iii) hospital-isolated, for cases confirmed as COVID-19 positive after being tested. The model was developed to incorporate the above scenarios:

$$\frac{dS}{dt} = -\beta(t)\frac{S}{N}\left(I_{ud,imp} + qQ_{I,imp} + I + qQ_I\right) \tag{7}$$

$$\frac{dE}{dt} = \beta(t)\frac{S}{N}\left(I_{ud,imp} + I + qQ_{I,imp} + qQ_I\right) - (\frac{1}{\tau} + \frac{1}{T_{qr}})E \tag{8}$$

$$\frac{dI}{dt} = \frac{1}{\tau}E - (\frac{1}{T_C} + \frac{1}{T_{qr}})I \tag{9}$$

$$\frac{dR}{dt} = \frac{1}{T_C}I \cdot (1 - p_1) \tag{10}$$

$$\frac{dQ_E}{dt} = \frac{1}{T_{qr}}E - \frac{1}{\tau}Q_E - \frac{1}{T_{hos}}Q_E \tag{11}$$

$$\frac{dQ_I}{dt} = \frac{1}{T_{qr}}E + \frac{1}{\tau}E - \frac{1}{T_C}Q_I - \frac{1}{T_{hos}}Q_I \tag{12}$$

$$\frac{dH_E}{dt} = \frac{1}{T_{hos}}Q_E - \frac{1}{T_{stay}}H_E \tag{13}$$

$$\frac{dH_I}{dt} = \frac{1}{T_{hos}} Q_I - \frac{1}{T_{stay}} H_I \tag{14}$$

$$\frac{dH}{dt} = \frac{1}{T_C} I \cdot p_1 - \frac{1}{T_{stay}} H \tag{15}$$

where S, E, I, Q_E, Q_I, R, H_E, H_I and H represent the number of individuals in different statuses, i.e. susceptible S, exposed E, infectious I, quarantined for exposed cases Q_E, quarantined (or self-isolated for infectious cases) Q_I, recovered R, hospital-isolated (for exposed cases after contact tracing and testing) H_E, hospital-isolated (for infectious cases after contact tracing and testing) H_I, and hospital-isolated (for cases that go to clinics or hospitals by themselves after symptom onset) H (Figure S1, Table S1). We set $p1$ as the probability of developing symptoms and hence going to a clinic or hospital. $1-p1$ is the fraction of asymptomatic cases, set as 0.17 [2]. We assumed that cases that recover without having been detected are asymptomatic cases. These cases are not quarantined, isolated at home (self-isolation) or isolated at hospital. The subscript '*imp*' refers to imported cases. Note that to simplify the notations, the variables without the label '*imp*' indicate the statuses of local cases. We refer to all infected individuals, generated by our model as 'cases'. Furthermore, $\beta(t)$ is the time-varying transmission rate, τ is the latent period and $\frac{1}{T_C}$ is the recovery rate (Please see Table S1 and Table S2 for the definitions of variables and parameters). Eqs. (2)-(6) describe the changes in the imported cases that can transmit disease to a local population (mainly from cases that were exempt from quarantine). Eqs. (7)-(15) describe the dynamics of local infections.

Some cases can be traced after a time interval, called **contact-tracing delay**. If they are contact-traced, exposed cases E can be quarantined as Q_E and infectious cases I can be isolated at home or quarantined as Q_I (depends on whether cases have developed symptoms). Our model did not explicitly distinguish between isolation at home (during symptomatic period) and quarantine (during pre-symptomatic transmission period) among Q_I since we assumed that their effects on the transmission are the same. Among quarantined or self-isolated cases, we assumed

that they are equally likely to infect household members with a contact rate adjusted by q, which is the ratio of the contact rates of quarantined to unquarantined individuals.

After a period of time waiting for testing results T_{hos}, referred to as **testing delay**, these contact-traced cases were confirmed and hospital-isolated as H_E or H_I as long as they tested positive.

In total, there are four types of confirmed cases among which contact-traced cases are labelled with an 'epi-link' (see section *Modelling the consequences of confirmation delay* in the main text):

1. **Contact-traced without confirmation delay**
2. **Contact-traced with confirmation delay**
3. **Backward contact-traced**
4. **No epi-link**

H_E refers to type 1 cases (Contact-traced without confirmation delay). H_I mainly result in type 2 cases (Contact-traced with confirmation delay) with a few type 1 cases that are confirmed during pre-symptomatic transmission period. A fraction ϕ of cases among H_I are confirmed with delay until after symptom onset, $H_{I,d}$. (To calculate ϕ and the proportion of traced cases with confirmation delay, see the section *Mapping model to cases with epidemiological links*). The whole confirmation time of cases in H_I is prolonged and is τ days longer, on average, than the confirmation time of cases in H_E. H denotes the status when cases are confirmed and isolated in hospital after they visit clinics or hospitals themselves. H can be further divided into H_B and H_{nl}. H_B refers to the cases that were back-traced with probability $p2$, hence with an epi-link, resulting in type 3 cases (Backward contact-traced). H_{nl} refers to cases without an epi-link, i.e. type 4 cases (No epi-link).

We further extended this model by incorporating the variations in contact-tracing delay and testing delay (see below). We are only interested in the hospitalized cases that require more intensive testing services and we assumed that the number of cases in hospital isolation affects the load on

testing mainly in the first week ($T_{stay} = 7$ days). Hence, in our model, H_E, H_I, H_B and H_{nl} actually reflect the number of hospital-isolated cases during the time when they place a higher demand on testing.

Incorporating variations in confirmation delay

$\frac{1}{T_{qr}}$ is the rate of contact tracing for both exposed and infectious cases. T_{qr} is the mean time interval between being exposed to a virus and the start of quarantine, also called **contact-tracing delay**. This delay is defined as the sum of the contact-tracing time and covert period (see Materials & Methods and Figure 1 in the main text). Contact-tracing time is determined by the capacity ($tshd_c$), load (i.e. the number of cases in quarantine or self-isolation), and the minimum ($mindelay_c$) and maximum delay ($maxdelay_c$) in contact tracing. The prior distribution of the minimum delay was adopted from a previous study of an outbreak that was successfully prevented during the 'first wave' in Hong Kong [5].

T_{hos} is the mean time interval between being quarantined (or self-isolation) and the confirmation of infection (hence the start of hospital isolation), also called **testing delay**. The value is determined by the capacity ($tshd_t$), load (i.e the number of cases in quarantine or self-isolation and in hospital isolation), and the minimum ($mindelay_t$) and maximum delay ($maxdelay_t$) of testing. The sum of contact-tracing delay and testing delay is the expected time between being exposed to a virus and being confirmed as a positive case. Note that the value of $mindelay_t$ was set to be half a day. The calculations of contact-tracing delay and testing delay were described in Materials & Methods in the main text. k, the factor to determine the contribution from the index case (covert period), was set to be 0.5.

For cases that were back-traced or cases without an epi-link, because most of them went to hospital/clinic after symptom onset, confirmation delay was referred to as testing delay, i.e. the time between symptom onset and confirmation of testing. We assumed that testing delay for these cases cannot be lower than the period of one generation time T_C minus the incubation time inc.

Incorporating effects of Tightening Social Distancing Measures

The time-varying transmission rate is calculated as $\beta(t) = C(t) * \beta_0$, where $C(t)$ is the relative contact rate and $\beta_0 = \frac{R_0}{T_C - \tau}$ (Table S2), is the baseline transmission rate. $C(t) = C_0$ is set to be the baseline of one before the relaxation of social distancing measures. $C(t)$ is updated after each social distancing measure is implemented. For example, $C(T)$ can be C_R (relaxation), C_{T1} (tightening 1), C_{T2} (tightening 2), P_{T3} (tightening 3) and C_{T4} (tightening 4), after the implementation of different social distancing measures.

During the relaxation of social distancing, we assumed that the increase of contact rate during the relaxation period followed a logistic curve. Because Tightening 1 (T1) imposed a policy similar to that before the relaxation of social distancing measures but a maximum occupancy of 60% of capacity was allowed in places of entertainment, compared with 50% before the relaxation, the prior of C_{T1} was set to be uniformly distributed between 0.95 to 1.2. To incorporate the effect of uncertainties in the timing of behaviour changes (*soc_unc*; see Table S3) after announcement of the interventions, we allowed the timing of $C(t)$ to be changed between 1.5 days before and 1.5 days after the implementation date of social distancing regulations. If *soc_unc* is negative, $C(t)$ decreases linearly starting from *soc_unc* days before until the actual implementation day. If *soc_unc* is positive, $C(t)$ begins to decrease *soc_unc* days after the actual implementation day.

During the relaxation period, $C(t)$ gradually increases following a logistic curve until the targeted C:

$$C(t) = C_0 + (C_R - C_0))\frac{1}{(1+exp^{\alpha(t-T_{50})})}$$

where α is a factor with negative value to determine the shape of the curve. T_{50} is the date with 50% of changes in C_R. *dur* was used in our model (Table S3) to indicate T_{50} for the relaxation of social distancing measures. C_0 is the baseline contact rate before relaxation of social distancing measures.

Mapping the model to cases with epidemiological links

We fitted the model's output simultaneously to reported (confirmed) lo-

cal cases both with and without an epi-link simultaneously. The joint likelihood of observing the numbers of daily confirmed local cases both with and without an epi-link was based on Poisson distributions with the means of the model-predicted numbers. The daily number of cases that were confirmed as cases with an epi-link each day was calculated as the increase of the sum of H_E, H_I and H_B by each day. The cases whose infection sources could not be be traced were confirmed as cases without an epi-link, H_{nl}. These model-predicted cases with an epi-link were compared with cases classified as 'Epidemiologically linked with local case'. Model-predicted cases without an epi-link were compared with cases that were classified as 'Local case' (without an epi-link) by the Hong Kong Centre for Health Protection (CHP) [3].

We compared the change in the actual proportion of contact-traced cases with confirmation delay with model predictions. The actual delay is calculated as $Reported\,date$ minus $Date\,of\,onset$ using the report from CHP. Since in our model, Q_I can include both cases during the pre-symptomatic transmission period and cases with symptoms, in order to estimate the daily number of new type 2 cases ($H_{I,d}$; contact-traced with confirmation delay) in H_I, we calculated the fraction of infectious cases among H_I that have transited from Q_I and have developed symptoms. The proportion was calculated as $\phi = exp^{-(\frac{delay}{\tau+\sigma})}$, where τ is the latent period, σ is the pre-symptomatic transmission period, the sum of them is the incubation time, and $delay$ is the expected confirmation time of each infected individual in H_I. This formula calculated the cumulative incidence of the event – symptom onset – until confirmation. The proportion of contact-traced cases with confirmation delay for each day is the daily change in $\frac{\phi H_I + H_B}{H_E + H_I + H_B}$.

Calculating effective reproduction number

The effective reproduction number, R_e, was calculated using the next-generation matrix approach after obtaining the posterior distributions of the model parameters [6]. The transmission matrix T and the transition matrix S were constructed. The elements in the first row of T represent the average number of newly infected cases in the exposed status transmitted by an exposed case (E); an infectious case (I); a quarantined

exposed case (Q_E); and a restricted infectious case (Q_I). Only I and Q_I can transmit the virus. The other rows represent the average number of newly infected cases in the infectious, quarantined exposed cases and restricted infectious cases, respectively. S represent the transition from cases in E, I, Q_E and Q_I (specified by the column) to cases in E, I, Q_E and Q_I (specified by the row). R_e was calculated as the first eigenvector of $-(TS^{-1})$ with the following formulas:

$$T = \begin{bmatrix} 0 & CR * beta * (\frac{S}{N}) & 0 & CR * q * beta * (\frac{S}{N}) \\ 0 & 0 & 0 & 0 \\ 0 & 0 & 0 & 0 \\ 0 & 0 & 0 & 0 \end{bmatrix}$$

$$S = \begin{bmatrix} -(\frac{1}{\tau} + \frac{1}{T_{qr}}) & 0 & 0 & 0 \\ \frac{1}{\tau} & -(\frac{1}{T_c} + \frac{1}{T_{qr}}) & 0 & 0 \\ \frac{1}{T_{qr}} & 0 & -\frac{1}{(\tau + T_{hos})} & 0 \\ 0 & \frac{1}{T_{qr}} & \frac{1}{\tau} & \frac{1}{T_{hos}} \end{bmatrix}$$

where the definitions of variables and parameters can be found in the section *SEIQR-D meta-population mode.*

Parameter estimation

The posterior distributions of the parameters of the SEIQR-D model for Hong Kong were obtained after fitting the model to the daily number of confirmed imported and local cases with symptom onset. The posterior distributions were estimated using a Markov chain Monte Carlo (MCMC) algorithm with 1.2×10^6 steps (Figure S2) to guarantee an effective sample size (ESS) of greater than 600 for all parameters.

Epidemiological parameters including the baseline transmission rate, latent period, pre-symptomatic transmission period, and generation time were set to be Gaussian distributed using the results adopted from a previous study (Table S2) [5]. Incubation time was calculated as the sum of latent period and pre-symptomatic transmission period, which was Gamma distributed. Prior distributions for all NPI-related parame-

ters were set to uniform distributions (Table S3), with the exception of the contact tracing and testing capacities ($thld_c$ and $thld_t$), minimum contact-tracing delay ($mindelay_c$), maximum confirmation delay ($maxdelay_c$ and $maxdelay_t$) and the ratio q, which were set to be Gaussian distributed based on previous studies or based on trial simulation results. Note that we set the differences between $mindelay_c$ and $maxdelay_c$ and between $mindelay_t$ and $maxdelay_t$ to be same to help MCMC trajectories to reach equilibrium. The Gelman-Rubin convergence diagnostic was used with two MCMC simulations to confirm these parameter estimates successfully reached convergence.

The generation time, defined as the average time interval between the infection time of an infected person and the infection time of the secondary infections, was implicitly assumed to be the sum of the infectious and latent period of an infected case in the model [7, 8]. The prior of the generation time was normally distributed with a mean of 7.5 days, using a previous study [9]. The prior of the ratio of the contact rates q also followed a normal distribution with a mean of 11% and a standard deviation of 0.1% from our previous study [5]. A recent study by Kwok et al. estimated that each person contacts an average of 12.5 others during a day [10]. Assuming many home-quarantined individuals are likely to have contact with individuals in their own household (expected number of 1.5 persons on average), the mean ratio of the contact rates of quarantined to unquarantined individuals in the prior distribution can thus be estimated approximately as $q = \frac{1.5}{12.5} = 12\%$.

The model derived mean maximum confirmation time was 10.9 days that occurred before targeted testing. The value was consistent to the previous observed reporting delay (10.3 days) during early spread period [11].

Model-fitting without variation in confirmation delay

We fitted the output from the reduced model (exactly the same model but excluding variation in confirmation delay) to daily reported local cases. The joint likelihood of observing the numbers of such cases both with and without an epi-link was based on Poisson distribution. Contact-tracing time was taken as 6 days and testing delay was 0.5 days. Amongst cases

visiting a clinic/hospital, the proportion that were back-traced was 25% (derived from posterior distribution of the full model). The posterior distributions were estimated using the MCMC algorithm with 1.2×10^6 steps to guarantee the convergence.

Supplementary Tables and Figures

Table S1: Descriptions of variables in the meta-population model.

**Variables**	**Descriptions**
E_{imp}	Exposed imported cases
Imp	Reported (confirmed) imported cases
$I_{ud,imp}$	Undetected infectious imported cases and some cases who did not comply with quarantine orders
$Q_{E,imp}$	Quarantined exposed imported cases
$Q_{I,imp}$	Restricted infectious imported cases (including both isolated and quarantined cases)
R_{imp}	Recovered imported cases
N	Population size in Hong Kong
S	Susceptible individuals
E	Exposed local cases
I	Infectious local cases
Q_E	Quarantined exposed local cases
Q_I	Restricted infectious local cases (including both isolated and quarantined cases)
H_E	Hospitalized exposed local cases
H_I	Hospitalized infectious local cases
$H_{I,d}$	Hospitalized infectious local cases with confirmation delay
H	Hospitalized cases that visits clinics or hospitals after their symptoms appear
H_B	Cases among H that were successfully back-traced later
H_{nl}	Cases among H that were not back-traced later.
R	Local cases who recovered without being detected

Table S2: Posterior estimates of reproduction numbers and other epidemiological parameters for COVID-19 transmission and control in Hong Kong. Mean values with 95% credible intervals are produced. The basic reproduction number R_0 is defined as the expected number of secondary infections without major NPIs, such as contact tracing, testing and quarantine, while maintaining baseline contact rate. The incubation time inc refers to the sum of the latent period and the pre-symptomatic transmission period $(\tau + \sigma)$. Note that the transmission rate β is not displayed because R_0 along with other parameters can determine β.

**Parameter**	Symbol	**Value** (95% **CI)**	**References**
Basic reproduction number (without quarantine measures and before relaxation of social distancing measures	R_0	2.36 (2.19 - 2.54)	[9,12,13]
Latent period (unit: days)	τ	1.90 (1.72-2.12)	[14]
Pre-symptomatic transmission period (unit: days)	σ	2.91 (2.74-3.09)	[15]
Incubation time (unit: days)	inc	4.81 (4.55 - 5.09)	[16,17]
Generation time (unit: days)	Tc	7.52 (7.33-7.72)	[9]

Table S3: Posterior estimates of NPIs parameters. Mean values with 95% credible intervals are produced. Note that contact rate before relaxation of social distancing measures was set to a baseline of one. Minimum testing delay, $mindelay_t$, was set to be 0.5 day.

**Symbol**	**Parameter**	**Value** (95% **CI)**
C_R	Relative contact rate of the relaxation of social distancing measures	2.97 (2.43 - 3.53)
C_{T1}	Relative contact rate of Tightening 1	1.14 (1.01 - 1.20)
C_{T2}	Relative contact rate of Tightening 2	0.98 (0.93 - 1.00)
P_{T3}	Protectiveness of face-mask wearing of Tightening 3	17.1% (1.8 - 36.0%)
C_{T4}	Relative contact rate of Tightening 4	0.79 (0.65 - 0.95)
dur	Required time for the relaxation of social distancing to achieve the maximum	14.2 (12.8 - 15.4)
$mindelay_c$	Minimum contact-tracing delay	3.89 (3.68 - 4.07)
$maxdelay_c$	Maximum delay in contact tracing	8.93 (8.65 - 9.20)
$maxdelay_t$	Maximum delay in testing	5.53 (5.35 - 5.71)
α	Rates of relaxation of social distancing measures to reach the maximum effects	-3.59 (-4.93 - -1.60)
κ_c	Capacity scale factor of contact tracing	9.5e-2 (3.8 - 14.7e-2)
$thld_c$	Capacity threshold of contact tracing (measured in case number)	20.19 (16.42 - 23.96)
κ_t	Capacity scale factor of testing	4.2e-3 (2.2 - 8.1e-3)
$thld_t$	Capacity threshold of testing (measured in case number)	19.85 (15.91 - 23.84)
$delay\_reduc1$	Reduction in Maxdelay from the first trace-and-test improvement (i.e. TT; see Table 1 in the main text)	53.9% (40.4 - 64.8%)
$delay\_reduc2$	Reduction in Maxdelay from the second trace-and-test improvement (i.e. IB; see Table 1 in the main text)	7.9% (0.3 - 23.5%)
soc_unc	Uncertainty of timing for social distancing becoming effective	-0.26 (-1.42 - 1.30)
$delay\_unc1$	Uncertainty of timing for the first improvement in tracing and testing (TT) to becoming effective	1.72 (0.07 - 3.68)
$delay\_unc2$	Uncertainty of timing for the second improvement in tracing and testing (IB) to becoming effective	1.66 (-2.63 - 4.78)
$p2$	Proportion cases visiting a clinic or hospital that were back-traced	24.4% (18.5 – 32.5%)
q	Ratio of the contact rates of quarantined to unquarantined individuals	11.4% (5.8 – 18.3%)

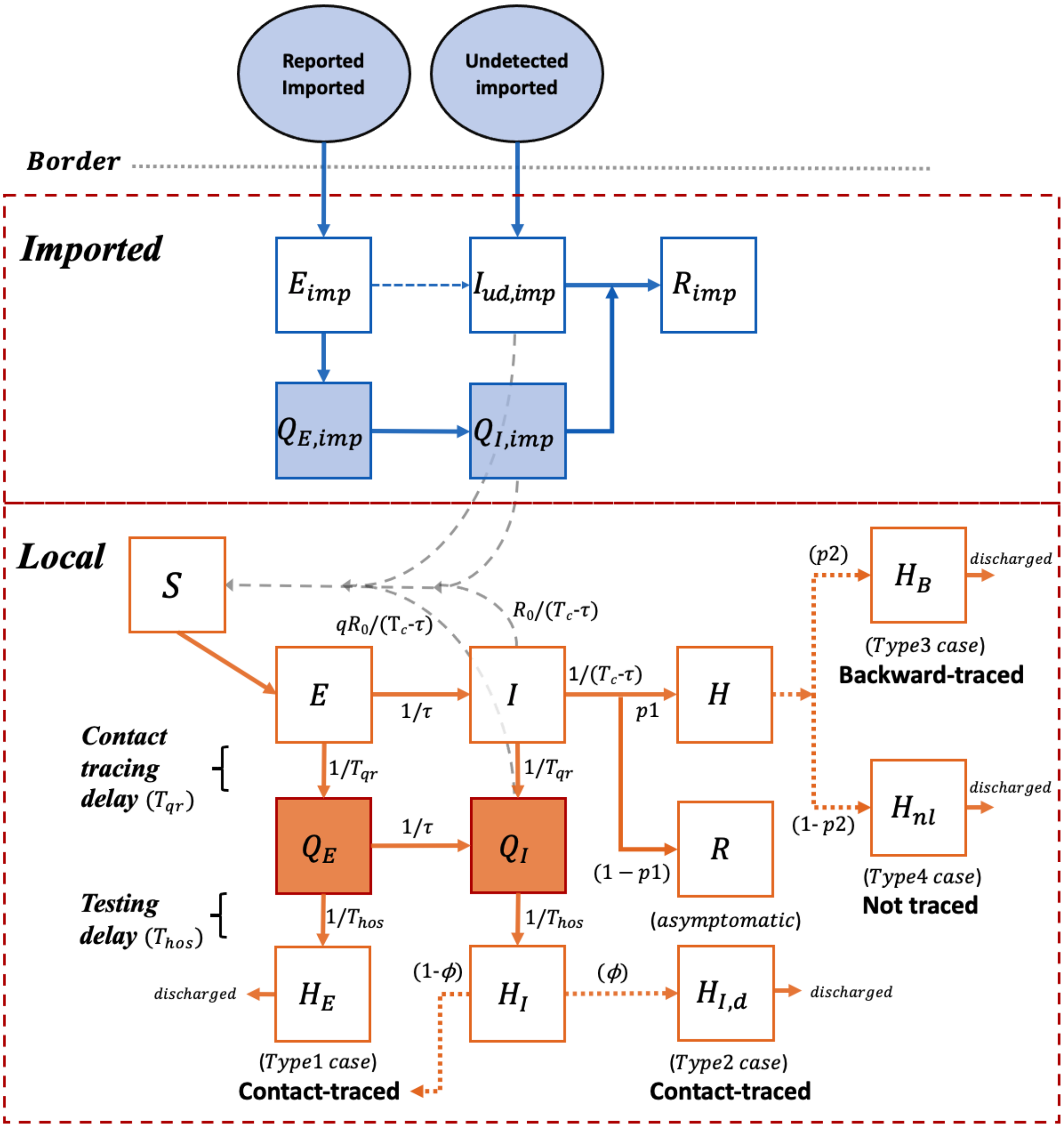

Figure S1: Susceptible-exposed-infected-quarantined-recovered-delay (SEIQR-D) metapopulation model with variations in confirmation delay. Top dashed box: Some imported cases arrive with exposed (E_{imp}) status before symptom onset, cross the border and then become, sequentially, quarantined during latent period ($Q_{E,imp}$), quarantined after latent period with infectiousness ($Q_{I,imp}$) and recovered (R_{imp}). Because of published accounts of occasional individuals who did not comply with quarantine orders, we assumed a small fraction (3%) of E_{imp} were not immediately quarantined (dotted line from E_{imp} to $I_{ud,imp}$). Bottom dashed box: Both undetected imported cases (plus few quarantined imported cases) and local infectious cases are able to infect susceptible individuals (S) and cause local (community) transmission. (Continued on the following page.)

Figure 1: (Continued) However, cases that are quarantined have a lower transmission rate than unquarantined cases, defined by the ratio of the contact rates of quarantined to unquarantined individuals q. τ is the latent period. T_{qr} is the time interval between becoming infectious and the start of quarantine (i.e. contact-tracing delay). T_{hos} is the time interval between being contact traced and being confirmed after testing (i.e. testing delay). Cases become hospital-isolated (H_E, H_I, H, H_B or H_{nl}) after confirmation until they are discharged. ϕ is the fraction of infectious cases confirmed and isolated as H_I after they have developed symptoms. $1 - p1$ is the fraction of asymptomatic cases. For the definitions of other variables and parameters, see text, Table S1, Table S2 and Table S3.

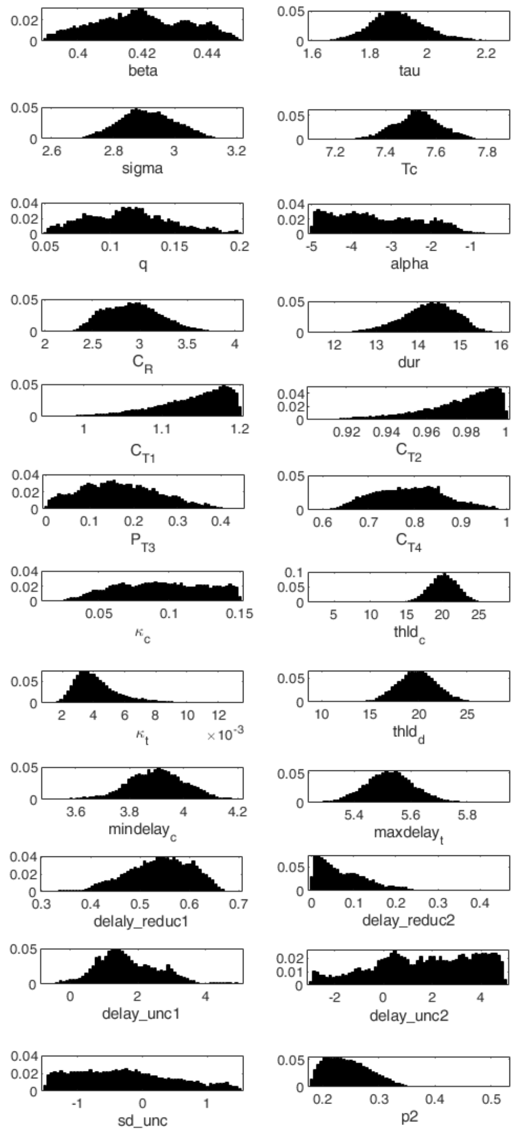

Figure S2: Posterior distributions of epidemiological parameters and the parameters of NPIs.

References

[1] Hsiang-Yu Yuan and Colin Blakemore. Suppression of an outbreak of COVID-19 without lockdown: the challenge of inefficient contact tracing and testing. (under review).

[2] Oyungerel Byambasuren, Magnolia Cardona, Katy Bell, Justin Clark, Mary-Louise McLaws, and Paul Glasziou. Estimating the extent of asymptomatic COVID-19 and its potential for community transmission: Systematic review and meta-analysis. *Official Journal of the Association of Medical Microbiology and Infectious Disease Canada*, 5(4):223–234, 2020.

[3] Hong Kong Centre for Health Protection. The latest situation of COVID-19 confirmed cases in HK (as of 25 October 2020). Available at https://www.chp.gov.hk/files/pdf/local_situation_covid19_en_20201025.pdf.

[4] Hong Kong Immigration Department. Statistics on Passenger Traffic. Available at https://www.immd.gov.hk/eng/message_from_us/stat_menu.html.

[5] Hsiang Yu Yuan, Guiyuan Han, Hsiangkuo Yuan, Susanne Pfeiffer, Axiu Mao, Lindsey Wu, and Dirk Pfeiffer. The importance of the timing of quarantine measures before symptom onset to prevent COVID-19 outbreaks - illustrated by Hong Kong's intervention model. medRxiv [Preprint] 2020. Available at https://doi.org/10.1101/2020.05.03.20089482.

[6] O. Diekmann, J. A. P. Heesterbeek, and M. G. Roberts. The construction of next-generation matrices for compartmental epidemic models. *Journal of the Royal Society Interface*, 7(47):873–885, 2010.

[7] Junling Ma. Estimating epidemic exponential growth rate and basic reproduction number. *Infectious Disease Modelling*, 5:129–141, 2020.

[8] J. Wallinga and M. Lipsitch. How generation intervals shape the relationship between growth rates and reproductive numbers. *Proceedings of the Royal Society B: Biological Sciences*, 274(1609):599–604, 2007.

[9] Qun Li, Xuhua Guan, Peng Wu, Xiaoye Wang, Lei Zhou, Yeqing Tong, Ruiqi Ren, Kathy S M Leung, Eric H Y Lau, Jessica Y Wong,

and Others. Early transmission dynamics in Wuhan, China, of novel coronavirus–infected pneumonia. *New England Journal of Medicine*, 382:1199–1207, 2020.

[10] Kin On Kwok, Ben Cowling, Vivian Wei, Steven Riley, and Jonathan M. Read. Temporal variation of human encounters and the number of locations in which they occur: A longitudinal study of Hong Kong residents. *Journal of the Royal Society Interface*, 15:20170838, 2018.

[11] M. Pear Hossain, Alvin Junus, Xiaolin Zhu, Pengfei Jia, Tzai Hung Wen, Dirk Pfeiffer, and Hsiang Yu Yuan. The effects of border control and quarantine measures on the spread of COVID-19. *Epidemics*, 32:100397.

[12] Adam J Kucharski, Timothy W Russell, Charlie Diamond, Yang Liu, John Edmunds, Sebastian Funk, Rosalind M Eggo, Fiona Sun, Mark Jit, James D Munday, Nicholas Davies, Amy Gimma, Kevin van Zandvoort, Hamish Gibbs, Joel Hellewell, Christopher I Jarvis, Sam Clifford, Billy J Quilty, Nikos I Bosse, Sam Abbott, Petra Klepac, and Stefan Flasche. Early dynamics of transmission and control of COVID-19: a mathematical modelling study. *The Lancet Infectious Diseases*, 20(5):553–558, 2020.

[13] Neil M Ferguson. Report 9: Impact of non-pharmaceutical interventions (NPIs) to reduce COVID-19 mortality and healthcare demand. Available at https://www.imperial.ac.uk/media/imperial-college/medicine/sph/ide/gida-fellowships/Imperial-College-COVID19-NPI-modelling-16-03-2020.pdf.

[14] Shujuan Ma, Jiayue Zhang, Minyan Zeng, Qingping Yun, Wei Guo, Yixiang Zheng, Shi Zhao, Maggie H Wang, and Zuyao Yang. Epidemiological parameters of coronavirus disease 2019: A pooled analysis of publicly reported individual data of 1155 cases from seven countries. medRxiv [Preprint] 2020. Available at https://doi.org/10.1101/2020.03.21.20040329.

[15] Wycliffe E Wei, Zongbin Li, Calvin J Chiew, Sarah E Yong, Matthias P Toh, and Vernon J Lee. Presymptomatic Transmission of SARS-CoV-2-Singapore. *Morbidity and Mortality Weekly Report*, 69(14):411–415, 2020.

[16] Wei-jie Guan, Zheng-yi Ni, Yu Hu, Wen-hua Liang, Chun-quan Ou, Jian-xing He, Lei Liu, Hong Shan, Chun-liang Lei, David S C Hui, Bin Du, Lan-juan Li, Guang Zeng, Kwok-Yung Yuen, Ru-chong Chen, Chun-li Tang, Tao Wang, Ping-yan Chen, Jie Xiang, Shi-yue Li, Jin-lin Wang, Zi-jing Liang, Yi-xiang Peng, Li Wei, Yong Liu, Ya-hua Hu, Peng Peng, Jian-ming Wang, Ji-yang Liu, Zhong Chen, Gang Li, Zhi-jian Zheng, Shao-qin Qiu, Jie Luo, Chang-jiang Ye, Shao-yong Zhu, and Nan-shan Zhong. Clinical Characteristics of Coronavirus Disease 2019 in China. *New England Journal of Medicine*, 382:1708–1720, 2020.

[17] Stephen A Lauer, Kyra H Grantz, Qifang Bi, Forrest K Jones, Qulu Zheng, Hannah R Meredith, Andrew S Azman, Nicholas G Reich, and Justin Lessler. The incubation period of coronavirus disease 2019 (COVID-19) from publicly reported confirmed cases: Estimation and application. *Annals of Internal Medicine*, 2020.
